# Creating a Measure of Shared Decision‐Making for Healthcare Decisions With Aboriginal and Torres Strait Islander People: A Hybrid Yarning and Modified Delphi Study

**DOI:** 10.1111/hex.70943

**Published:** 2026-10-27

**Authors:** Marguerite Tracy, David Follent, Emma Webster, Tara Dimopoulos‐Bick, Lyndal J. Trevena, Melissa Cawley, Michelle Dickson, Heather L. Shepherd

**Affiliations:** ^1^ General Practice Clinical School, Faculty of Medicine and Health, The University of Sydney Sydney NSW Australia; ^2^ Sydney Health Literacy Lab, Faculty of Medicine and Health, The University of Sydney Sydney NSW Australia; ^3^ Department of General Practice Faculty of Health, Western Sydney University Sydney NSW Australia; ^4^ Aboriginal Chronic Conditions Network, Aboriginal Health and Wellbeing, Agency for Clinical Innovation, St Leonards St Leonards NSW Australia; ^5^ School of Rural Health, The University of Sydney Sydney NSW Australia; ^6^ The Poche Centre for Indigenous Health, The University of Sydney Sydney NSW Australia; ^7^ Susan Wakil School of Nursing and Midwifery, The University of Sydney Sydney NSW Australia

**Keywords:** Aboriginal and Torres Strait Islander, patient involvement, questionnaires, shared decision making

## Abstract

**Objectives:**

Shared decision making (SDM) is a process which involves an exchange of information between healthcare professionals and patients about options (including watch and wait) and the patient's preferences. It is recognised as an important part of person‐centred health care and is part of national standards for delivery of care in Australia. SDM improves health outcomes and SDM resources specifically designed with and for Aboriginal and Torres Strait Islander communities are increasingly available. However, there is no culturally appropriate measure to evaluate the SDM process and the impact of these resources. The objective was to create a culturally safe measure of SDM for Aboriginal and Torres Strait Islander people and communities using the nine‐item SDM‐Q‐9 as a starting point.

**Methods:**

A purposively invited expert panel of Aboriginal and Torres Strait Islander Health Professionals and Community members and experts in SDM attended a hybrid yarning briefing session incorporating yarning (a cultural approach to dialogue). The panel then participated in a two round online survey Delphi process. Study team members reviewed the yarning session, developed themes to apply to questions for the Delphi rounds. Team members analysed and reported results of each round to participants and proposed the final measures based on feedback in each round.

**Results:**

Eighteen of 26 invited participants attended the yarning briefing session and 14 participated in both Delphi rounds. Consensus was reached for a measure with 12 items, their wording and the cultural concepts supporting each. The resulting measure included new wording for all items and the addition of three items. From the yarning session the need for a short measure resulted in a proposed five‐item version of the measure with the basic aspects of SDM was also agreed on in the Delphi process. Both measures will continue for next stage testing.

**Conclusions:**

Using a hybrid yarning and Delphi method resulted in a culturally adapted measure of SDM. Validation and real‐world testing of the measure are planned for the 12‐ and 5‐item version created.

**Practice Implications:**

Once validated, this co‐created culturally responsive measure will be used to assess the efficacy of processes and tools to support shared decision making with Aboriginal and Torres Strait Islander people.

**Patient or Public Contribution:**

An Aboriginal and Torres Strait Islander Governance Group provided oversight and guidance throughout the project, ensuring it was shaped by Indigenous knowledge, cultural protocols, and ethical principles. The Group emphasised reciprocal learning, respect for diverse knowledge systems, and the use of Aboriginal and Torres Strait Islander research methods. It supported community benefit, collaborative cultural approaches, and Indigenous‐led decision‐making. The Group also ensured findings were prepared and shared in culturally safe ways, recognising knowledge ownership and appropriate dissemination. Additionally, it advised on future directions, strengthening the project's ongoing impact while maintaining alignment with Aboriginal and Torres Strait Islander values, priorities, and processes. Governance group chairs were observers at the yarning session and no Governance Group members were part of the Delphi process.

## 1 Background

Shared decision making (SDM) is a process where patients and healthcare providers make informed and preference‐based health decisions together [1, 2]. SDM is part of Australian National Safety and Quality Health Service standards and has been shown to improve health outcomes for people [3, 4]. SDM may be one strategy to help address ongoing health and life expectancy inequities for Aboriginal and Torres Strait Islander people [5]. There is a vast array of tools to support SDM with an increasing number being created with and for Aboriginal and Torres Strait Islander people (First Nations) in Australia [6, 7]. One such tool was co‐designed with Aboriginal people in NSW Australia originally to support SDM around COVID‐19 vaccination called Finding Your Way (https://aci.health.nsw.gov.au/shared-decision-making) [8].

Finding Your Way is a non‐linear, interactive model which has eight circles representing core elements or ‘concepts’ of a cultural perspective of SDM including ‘Options’, ‘Take a break’, ‘Feeling safe’ and ‘Knowing, being and doing’. The development of Finding Your Way and the supporting resources was led by the New South Wales (NSW) Agency for Clinical Innovation in collaboration with Aboriginal Health Workers and community members living and working on Eora, Wilyakali, Gandangara, Wiradjuri, Bundjalung, Darug and Yuin lands in NSW during 2021. It uses Aboriginal and Torres Strait Islander pedagogies such as the Aboriginal 8 Ways of Learning [9]. The involvement and leadership of Aboriginal people in the development of Finding Your Way provided valuable information and perspectives to maximise feasibility and acceptability [10].

With a culturally responsive SDM model and resources co‐designed and available, a measure that captures all included domains is now required to enable evaluation of adoption of SDM, the reported experience of SDM and the impact of SDM in practice. There are many internationally recognised measures of SDM [11, 12] such as SDM‐Q‐9, a nine‐item questionnaire which provides a score quantifying core components of SDM in practice [13], but this does not include the additional cultural elements of sharing medical decisions with Aboriginal and Torres Strait Islander people as highlighted in the Finding Your Way model [10]. SDM‐Q‐9 has been translated into several languages but has never been culturally adapted [14]. Whilst culturally adapted tools to support SDM have been reported we were unable to find a culturally adapted measure certainly none co‐designed with Aboriginal and Torres Strait Islander people. This study aimed to address this gap using elements of content validation and conceptual equivalence in measure development using expert and community consensus [15].

## 2 Aim

The objective was to create a culturally safe measure of shared decision making for Aboriginal and Torres Strait Islander people and communities using the nine‐item SDM‐Q‐9 as a starting point [13, 14].

## 3 Methods

### 3.1 Permissions

Permission from the original developers to culturally adapt SDM‐Q‐9 was sought and granted.

### 3.2 Ethics and Governance

Ethical approval was granted by both the Aboriginal Health and Medical Research Council of NSW (NSW AH& MRC) (2212/24) and The University of Sydney Research Ethics Committee (2023/HE000754). An Aboriginal and Torres Strait Islander Governance Group provided oversight and guidance throughout the project, ensuring it was shaped by Indigenous knowledge, cultural protocols, and ethical principles. The Group emphasised reciprocal learning, respect for diverse knowledge systems, and the use of Aboriginal and Torres Strait Islander research methods. It supported community benefit, collaborative cultural approaches, and Indigenous‐led decision‐making. The Group also ensured findings were prepared and shared in culturally safe ways, recognising knowledge ownership and appropriate dissemination. Additionally, it advised on future directions, strengthening the project's ongoing impact while maintaining alignment with Aboriginal and Torres Strait Islander values, priorities, and processes.

### 3.3 Study Design

The development of the measure was achieved using an initial yarning briefing session, analysis to create a survey and two rounds of a modified Delphi process [16] conducted between June and December 2024 by a collaboration between NSW Agency for Clinical Innovation and the University of Sydney. The process was overseen by the Aboriginal and Torres Strait Islander Governance Group.

Prospective expert Delphi panel members were recruited via general and/or personalised email from the research team using a purposive and convenience sampling approach through existing research, organisational and personal cultural networks. This approach ensured national membership of Aboriginal and Torres Strait Islander experts with an interest in SDM and researchers with expertise in SDM across Australia and internationally. No external consulting regarding the Delphi method took place.

#### 3.3.1 Panel Selection Criteria

Inclusion criteria for the expert Delphi panel required participants to be over 18 years of age and to have expertise in at least one relevant area. This included being an Aboriginal Health Worker or Aboriginal Health Practitioner in Australia, an Aboriginal and Torres Strait Islander community member or leader, having experience working in or with Aboriginal Community Controlled Health Services, or be involved in SDM research. In addition, participants needed knowledge or expertise in supporting people to make decisions about their health care (SDM), measuring SDM, or the use or design of patient‐reported outcome and experience measures (PROMs/PREMs).

The sampling matrix for the expert panel was developed and guided by the Aboriginal and Torres Strait Islander Governance Group. The aim was for the panel to represent a balance in membership across gender, health professional and community member representation, and areas of expertise. This balance was important to ensure the focus of this study remained as cultural adaptation of the tool for Aboriginal and Torres Strait Islander people. Previous research has shown that impartiality enhances the validity of Delphi results and as such participants did not need prior knowledge of specific SDM measurement tools or Finding Your Way [17]. Prospective panel members were invited to participate in the Delphi study and attend a yarning briefing session prior to the survey rounds.

#### 3.3.2 Yarning Briefing Session

The study used a hybrid yarning briefing session (online and in person facilitated via Zoom) incorporating yarning methods [18]. Yarning methods privilege cultural safety of participants; the research team opened the discussion and asked questions then listened in to the story/discussion which ensued [18]. The yarning briefing session was followed by a two round Delphi process, with additional rounds if required, to create a culturally responsive measure of SDM initially based on an existing measure. This approach had been used to gather expert opinions and reach a consensus on the capabilities required to effectively use Finding Your Way [8].

The yarning briefing session was planned as a two‐way learning process to describe and yarn about concepts of SDM (see Box 1), measurement of SDM, cultural aspects of SDM, with some information shared with participants about Finding Your Way and SDM‐Q‐9 prior to the session. Notes from this interaction were used to design the Round 1 survey of the Delphi process.

Box 1 **SDM concepts.**

Feeling safe, Feeling trusted, Sharing your truths, Sharing your stories, Decisions, Options, Preferences, Involvement, Understanding.

The 2‐h Delphi panel yarning briefing session was held in April 2024 facilitated by three research team members (HS, DF and MT) and was held in hybrid mode with groups and individuals joining from around Australia and internationally. Permission to record the session was granted for note taking purposes. The session was recorded (audio only) to ensure details of the discussion were captured in full and able to inform the Delphi survey development. HS and MT reviewed the session and created the Round 1 survey based on feedback and themes from the session.

### 3.4 Survey Rounds

The Delphi survey was created in REDCap, an electronic data capture platform [19, 20] with one item per page. Panellists were sent an email invitation to complete the survey online. Rounds were planned until the a priori defined consensus of 75% ‘Agree’ or ‘Strongly agree’ was reached on all elements of the final measure. Panellists were required to rate agreement for each statement on a 5‐point Likert scale (strongly disagree, disagree, neutral, agree, strongly agree). Each statement was mapped to the concepts in Finding Your Way based on previous work [10] and was assessed by (1) agreement by participants on alignment to the core cultural concepts from Finding Your Way and (2) agreement on the wording of the statement. Participants had the opportunity to add further comments for each statement (Box 2). We anticipated two or three rounds would be required to achieve the desired consensus, further rounds were possible if needed. Each survey was sent by email to the panellists, with a reminder sent two weeks later. On completion of the study panellists were offered gift cards acknowledging their time and expertise.

Box 2 **Format of statements in Delphi survey.**

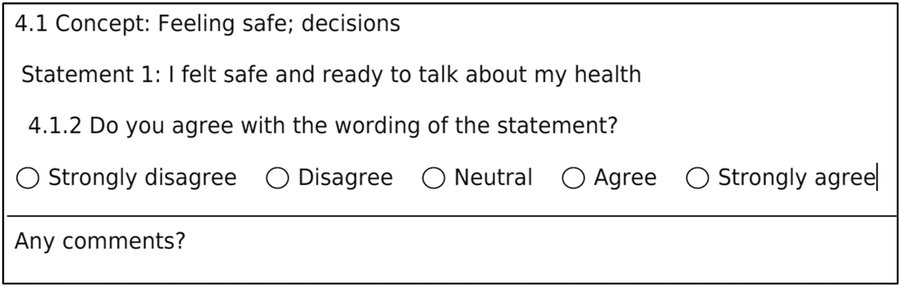

### 3.5 Data Analysis

Quantitative data and additional qualitative comments were exported to Microsoft Excel for analysis. Analysis was undertaken by HS and MT with each round reported to the research team for feedback and discussion. A summary of the initial yarning briefing session was shared with participants in Round 1, subsequently, results of Round 1 were distributed to participants integrated into the Round 2 survey. Results were shared and discussed with the Aboriginal and Torres Strait Islander Governance Group.

## 4 Results

Twenty‐six people were invited to participate in the expert panel. The yarning briefing session was attended by 17 people, of which 12 attendees went on to be Delphi panellists (*n* = 12) The 17 attendees included Aboriginal and Torres Strait Islander health experts and Community members (*n* = 10) and local and international researchers with expertise in measurement of SDM (*n* = 2). In addition, the two co‐chairs of the Aboriginal Governance Group for the project attended (*n* = 2) as well as three members of the research group who facilitated the session (*n* = 3). The session was not compulsory for participation in the Delphi process, but it was designed to provide additional information and initial discussion to enable additional panellists from around Australia and internationally to take part in the Delphi rounds, two panellists were unable to attend the yarning briefing session. All Delphi panellists had access to the yarning briefing session slides, notes and resources prior to the survey rounds. Fourteen expert panellists took part in two rounds of the Delphi Survey study between August and October 2024. All panellists (*n* = 14) completed both rounds. Panellist demographics and areas of expertise are listed in Table 1.

**Table 1:** Delphi expert panel information.

Characteristic	N in panel*
Aboriginal or Torres Strait Islander	
Aboriginal	6
Torres Strait Islander	1
Both Aboriginal and Torres Strait Islander	2
Neither Aboriginal nor Torres Strait Islander	5
Self‐reported area of expertise*	
Aboriginal Health Worker or Health Practitioner	5
Aboriginal and/or Torres Strait Islander Community leader	3
Experience working in or with an Aboriginal Community Controlled Health Service	6
Experience helping people make decisions about their health care (SDM)	8
Expertise in measuring SDM	5
Expertise in the use, or design of patient reported outcome/experience measures (PROMs/PREMs)	3
Healthcare consumer	3
Australian State or Territory of work	
Australian Capital Territory	1
New South Wales	7
Northern Territory	1
Queensland	3
South Australia	1
Tasmania	1
Victoria	1
Western Australia	0
Not worked/working in Australia	2

* Total panel *n* = 14. Note some categories multiple selection possible.

### 4.1 Summary of the Yarning Briefing Session Discussion

The yarning briefing session started with an introduction to the research, *Finding Your Way*, SDM and the rationale for a culturally adapted SDM measure. General discussion included how cultural difference influenced a conceptual view of SDM such as the importance of two‐way conversation in decision making and the need for health professionals not to make assumptions about knowledge or decision preferences. Implementation aspects of the proposed tool were also discussed such as ensuring healthcare professionals being oriented to local culture, ensuring implementation of measures to support SDM were appropriate and supported improving health literacy and the impact of jargon particularly when not explained or used appropriately.

The attendees discussed supporting the journey of all stakeholders through implementation through sharing information, informing decisions and choices and empowering patients and communities. The group noted that the new SDM measure was highly likely to be used by services within future quality improvement projects.

The SDM‐Q‐9 measure was shared with the attendees as a starting point for discussion and to illustrate what is typically included in measures of SDM. This enabled attendees to reflect on what may be missing or what may be redundant.

Specific points raised by the group to consider for inclusion in a culturally adapted SDM measure were incorporated into the Round 1 survey, these were:

− The measure needed to include a statement which explored whether the participants (healthcare professionals and patient) felt culturally safe to discuss health and decisions surrounding it.
− Ensuring the measure captured whether the person could include support from family and community was important.
− The measure needed to include a statement that explicitly referred to allowing time for decision making.

Additional points discussed by attendees included: the length of the measure, that it was about right but a short version would be valuable in some circumstances; terminology for the healthcare provider (SDM‐Q‐9 uses ‘doctor’), as discussions are frequently with Aboriginal Health Workers and other clinicians; and a preference for use of the active voice.

Following the yarning briefing session the Round 1 survey was developed to include elements from the discussion and to reach consensus on several aspects of the proposed measure. Twelve proposed statements were created by the study team to be presented to panellists in Round 1 of the Delphi. Panellists were asked to reach agreement on: the terminology used in the statements in the proposed measure; the preamble wording to introduce the measure and the question regarding the health decision context in the healthcare encounter; whether each proposed SDM measure statement matched the mapped concept; the wording of each SDM measure statement and the response items; and the length of the measure.

### 4.2 Summary of Round One

A total of 14 panellists completed Round 1 with 75% or above agreement reached for the following:

- It is important to ask about the decision which was made in the encounter with the healthcare professional (93% agreement, 13/14).
- The wording asking about the healthcare encounter reason and decision made (79% agreement, 11/14).
- The response items to ask about agreement with the SDM measure statements (86% agreement, 12/14).
- 8 of the 12 possible SDM measure statements reached 79% (11/14) or higher agreement, for both the concept mapping the statement and the wording of the statement.

Comments were independently reviewed by two team members (HS and MT) who discussed inclusion for Round 2. There were no points of contention arising from the comments made in Round 1. Two topics raised in the comments were focussed on the term used for the healthcare professional (see Table 2) and the term ‘my’ (raised by one panellist) when referring to the healthcare provider. These remaining issues were also carried forward to Round 2 to seek agreement on: the introduction wording; the wording of the instructions for the measure; and including a definition for a preferred term for the healthcare professional: *The SDM‐Q‐9 tool uses the term ‘my doctor’. How satisfied are you with replacing this with ‘the health worker’ with the addition of the description in the instructions? “There are 11 sentences below about your yarn/discussion with the health worker (Aboriginal health worker/doctor/nurse etc.). For each sentence, please tick a box to show how much you agree or disagree.”*

**Table 2:** Suggested terms for the healthcare professional.

Term for healthcare professional	*N*
My healthcare worker	5
My healthcare provider	5
My doctor	2
My healthcare professional	2

The expert panel were also asked to share their preferred length for a final measure. Four experts selected the option ‘Unsure’, two selected ‘1–5 questions’, the remainder (*n* = 8) opted for ‘6–10 questions’ with no participants choosing ‘11–15 questions’ as an ideal length for the measure. In the free text one participant suggested that a short version of the measure be created. In response to this feedback HS and MT proposed the 5‐item measure taking into account the themes of the yarning briefing session and the core constructs of SDM. The proposed 5‐item measure was proposed in the Round 2 survey seeking agreement with the five proposed items and noting that further validation of the short version would be undertaken later.

### 4.3 Summary of Round Two

A total of 14 panellists completed Round 2, a 100% completion rate between both rounds. Round 2 presented amended wording for five of the eight statements that had reached consensus in Round 1 and re‐presented the four amended statements that did not reach agreement in Round 1. Feedback from the first round was presented to panellists with the summary of Round 1 results and second round questions (Box 3).

Box 3 **Example of feedback to participants in round 2.**

2.4 We reached consensus on the following item:

I talked about/discussed the advantages and disadvantages of the options with my healthcare worker

Suggested wording change:

*I talked about the advantages and disadvantages of the options with my healthcare worker*

To what extent are you comfortable with this suggested wording change?

The five statements that reached consensus in Round 1, were presented again in Round 2 with wording clarity improved from panellists’ comments. As a result, consensus was sought for the amended wording in Round 2 (Table 3).

**Table 3:** Wording suggestions from Round 1 reaching consensus in Round 2.

Round 1 wording	Round 2 suggested wording	Consensus (%, *n*/14)
I talked about/discussed the advantages and disadvantages of the options with my healthcare worker	I talked about the advantages and disadvantages of the options with my health worker	93%, 13/14
I talked about/discussed my goals and concerns about the options with my healthcare worker	I talked about my goals and concerns about the options with my health worker	93%, 13/14
I felt able to take a break or not do anything right now	I felt able to take a break or choose not to do anything right now	100%, 14/14
I was able to share what option I prefer	I felt able to share the option I prefer	86%, 12/14
I chose the treatment I prefer	I felt able to choose the best option for me	79%, 11/14

Round 2 sought consensus for the four statements that did not reach agreement in Round 1 for either alignment with the SDM mapped concepts, wording or retention of the statement in the final measure. Consensus (75% strongly agree) was reached for 3 of the 4 statements presented (Table 4).

**Table 4:** Round 2 Consensus.

Round 1	Round 1 outcome (%, *n*/14) with further comments	Round 2 suggested wording	Consensus (%, *n*/14) and further feedback for implementation
Concepts: Feeling safe; Involvement (of person and others). Statement 2: I was invited to make a decision with my healthcare worker.	Consensus on concept matching the statement: 86%, 12/14 Wording: 71%, 10/14 Comments included replacing the word ‘invited’ to asked, and whether the statement reflected the notion of safety.	The health worker supported me to make a decision about my health with them.	93%, 13/14
Concepts: Options, Understanding. Statement 5: I knew/learned that there are a number of options to treat my health care problem	Consensus on concept matching the statement: (93%, 13/14) Wording: (64%, 9/14) Comments included replacing the word learn with ‘understand’. The literacy level of the statement was also considered.	I understand there are options to treat my health care problem	93%, 13/14
Concepts: Sharing your stories, Understanding. Statement 8: I had time to understand the health care options.	Consensus not reached on the statement matching the concept (71%, 10/14) Wording (79%, 11/14) Comments noted that the statement aligned better with ‘Take a break’ or ‘Sharing Stories’. The concepts are covered in 2 other statements. Statement 7: I talked about/discussed my goals and concerns about the options with my healthcare worker Statement 9: I felt able to take a break or not do anything right now. We suggest removal of the item as the concepts are not well matched and are covered by Statements 7 and 9.	I felt able to share the option I prefer	(71%, *n* = 10/14) Consensus was not reached to remove this statement
Concepts: Feeling safe, Understanding, Decisions. Statement 12: I think we chose a good option.	Consensus on concept matching the statement: (86%, 12/14) Comments did not suggest any improvements, except for opposing views of the use of ‘we’, one panellist liking the use of ‘we’, another wanting ‘we’ to be removed. Another comment was made about the use of ‘I think’ suggesting uncertainty.	I felt we chose the best option	86%,12/14 ‘I felt we chose the best option for me’ suggested in this round will be further tested in implementation

The Round 2 survey also resulted in consensus at 86% 12/14 or higher for the context and instruction statements, ‘Do you think it is important to ask about the decision made?’, ‘Why did you visit your healthcare worker today?’ and ‘What health issue did you talk to them about today?’

At the end of Round 2, consensus (75% strongly agree) was reached for 11 of the 12 statements. Agreement with the proposed 11‐item measure was 93%, (13/14).

Similarly, consensus (93%, *n* = 13/14) was reached for a 5‐item version of the tool to move forward to validation and implementation testing.

For both versions, one panellist commented that they were neutral given their reservation about the broader concepts of cultural safety (Statement 1) being challenging to capture in a survey.

Consensus was not achieved for the term that should be used to describe the patient in the healthcare provider version of the tool (71%, *n* = 10/14 for the term ‘patient’). All participants who identified as a healthcare professional in the survey agreed with the use of ‘patient’. This term will be retained as no other suggestion which was clear was made e.g. the person was potentially too ambiguous in this context, noting also that this version of the form is not seen by the patient, only the healthcare professional in the interaction. The term ‘patient’ will be tested further in validation and implementation.

### 4.4 Final Measure and Suggestions Provided for Implementation

Figure 1 shows the final agreed cultural adapted measure of SDM which will be validated and used in implementation. The full version of the agreed measure includes all 12 statements, as removal of Statement 8 did not reach consensus. The acceptability and function of this statement will be tested during future validation.

**Figure 1:**
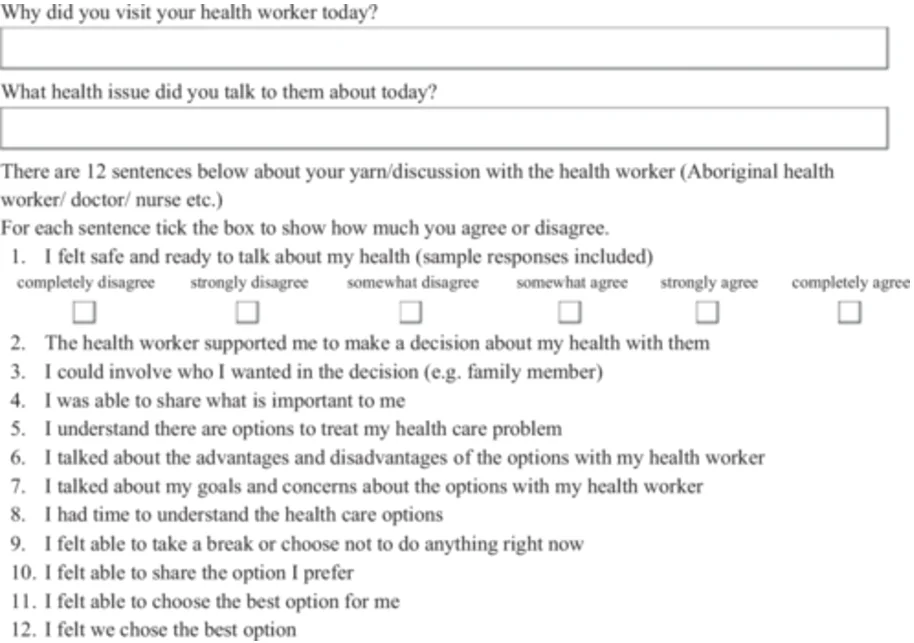
Final consensus approved measure.

The 5‐item version will also be validated in further planned testing (Box 4).

Box 4 **5‐item version.**

1. I felt safe and ready to talk about my health.
2. I could involve who I wanted in the decision (e.g. family member).
3. I understand there are options to treat my health care problem.
4. I talked about my goals and concerns about the options with my health worker.
5. I felt able to choose the best option for me.

## 5 Discussion

This study used a yarning session followed by a two‐round modified Delphi study, resulting in the first known cultural adaptation based on the SDM‐Q‐9 measure of SDM for use in healthcare consultations with Aboriginal and Torres Strait Islander people. Using two‐way learning, overseen by Aboriginal and Torres Strait Islander Governance, a panel of experts reached consensus agreement on essential item statements, their wording and the underlying cultural concepts of SDM. The new measure incorporated three further concepts which arose from the yarning briefing session: feeling culturally safe to participate in making a decision; the ability to involve others in the decision; and an explicit statement of having time to make the decision. This resulted in a longer measure and the need to test the validity of a shorter measure (which does not include a separate item for time). The wording of statements was altered to reflect the personal experience using ‘I’ statements and simplified the language where possible and to reflect the provision of healthcare in the Australian setting. During the process it became clear that the measure would be unlikely to remain a nine‐item measure and it was discussed that the measure developed in the process may not be described as a cultural adaption of SDM‐Q‐9 but may be a new measure inclusive of the concepts in that measure.

A 12‐item measure and a brief 5‐item version of the measure reached consensus. The panel supported proceeding to validation and implementation feasibility testing of both versions.

### 5.1 Aligning Culturally Adapted Models and Measures of SDM

This study provides an opportunity to strengthen the cultural meaning of SDM and consequently to more fully reflect whether this approach is experienced by people, family, community and kin. The measure resulting from this work will be used to quantify bringing these two elements together. The *Finding Your Way* model of SDM has incorporated the strengths of Aboriginal culture with constructs of SDM, self‐determination and community engagement [10]. Measure development and adaptation to culture and community includes attention to content relevance and coverage, and conceptual equivalence, ensuring that people completing measures see the concept in the same way [15, 21]. When implementing programs and resources to support SDM with Aboriginal and Torres Strait Islander peoples it is imperative that these incorporated strengths and culturally important constructs and concepts are aligned and accurately covered in a validated measure of SDM.

### 5.2 Readiness as a Culturally Specific Construct Necessary for SDM

A key point that was identified by Aboriginal and Torres Strait Islander community members in the yarning briefing session was establishing readiness to participate in a process of SDM and feeling safe to make decisions with the healthcare professional. Evidence of the determinants for readiness has been more recently explored both generally [22] and for specific healthcare decisions [23] with a measure for readiness for SDM recently developed [24]. Measures like this will also need to be evaluated for use with Aboriginal and Torres Strait Islander people and potentially culturally adapted for different local and community contexts. Our findings underlined this as an important concept for people to comfortably participate in decision making. As such the first statement in our measure, makes these concepts clear ‘I felt safe and ready to talk about my health’. Readiness and safety emerged in our discussions as highly important for Aboriginal and Torres Strait Islander people, and this may well resonate with people from all cultures.

### 5.3 Integrating Cultural Safety Into Measures of Safety and Quality

SDM and cultural safety are important components of high quality and safe care, explicitly stated in international health care standards (in Australia, New Zealand and Canada) [3, 25, 26]. Capturing the experience of people in health encounters through validated measures is an important step in identifying whether services are providing care that aligns with and meets expected standards, values and preferences. Measures that are culturally safe and aligned to cultural understanding and constructs will provide data that can inform quality improvement initiatives and required training programs to support health services [27].

#### 5.3.1 Strengths and Limitations

Fourteen panellists brought diverse expertise to the Delphi panel from research, healthcare, Aboriginal and Torres Strait Islander cultural knowledge and experience, consumer experience and SDM however no experts had all these backgrounds. A larger panel may have added strength to the consensus conclusions drawn from the rounds conducted. After two rounds there were items in the survey which did not reach consensus, and the decision was made to use the existing expert panel input and explore these areas further in later steps of validation and implementation.

## 6 Conclusions

Using a yarning briefing session and modified Delphi study method, a cultural adaptation of a measure of SDM based on SDM‐Q‐9 has been created. This new measure enables measurement of SDM that not only reflects Aboriginal and Torres Strait Islander cultural values but also centres the experience of SDM by the person in health care consultations. A 12‐item and 5‐item tool reached consensus for use in future validation studies and to measure implementation of healthcare initiatives which support SDM with Aboriginal and Torres Strait Islander people.

## Author Contributions

**Marguerite Tracy:** conceptualisation, investigation, funding acquisition, writing – original draft, methodology, writing – review and editing, formal analysis, project administration, data curation, resources. **David Follent:** conceptualisation, writing – review and editing, methodology, investigation, resources. **Emma Webster:** conceptualisation, investigation, writing – review and editing, methodology. **Tara Dimopoulos‐Bick:** conceptualisation, investigation, methodology, writing – review and editing. **Lyndal J. Trevena:** conceptualisation, investigation, supervision, writing – review and editing, methodology. **Melissa Cawley:** conceptualisation, investigation, methodology, writing – review and editing. **Michelle Dickson:** conceptualisation, writing – review and editing, supervision. **Heather L. Shepherd:** conceptualisation, investigation, funding acquisition, writing – original draft, writing – review and editing, methodology, data curation, project administration, resources, formal analysis.

## Conflicts of Interest

The authors declare no conflicts of interest.

## Data Availability

The data that support the findings of this study are available from the corresponding author upon reasonable request.
